# M6a is expressed in the murine neural retina and regulates neurite extension

**Published:** 2008-09-03

**Authors:** Jing Zhao, Atsumi Iida, Yasuo Ouchi, Shinya Satoh, Sumiko Watanabe

**Affiliations:** Department of Molecular and Developmental Biology, Institute of Medical Science, University of Tokyo, Tokyo, Japan

## Abstract

**Purpose:**

Glycoprotein m6a (M6a) is a cell-surface glycoprotein that belongs to the myelin proteolipid protein family. M6a is expressed mainly in the nervous system, and its expression and function in mammalian retina have not been described. Using proteomics analysis of mouse retinal membrane fractions, we identified M6a as a retinal membrane protein that is strongly expressed at embryonic stages. Our aim was to reveal the function of M6a in development of mouse retina in this work.

**Methods:**

Detailed expression pattern of M6a was examined by immunostaining using frozen sections of mouse retina obtained at various developmental stages. For functional analysis of M6a in mouse retinal development, we performed retorovirus-mediated overexpression of M6a in mouse retinal explant culture. Then, cell differentiation, proliferation and structural maturation of the cells were examined.

**Results:**

M6a transcripts were strongly expressed in embryonic retina. After completion of retinal differentiation, the level of expression decreased as mouse development progressed. Immunohistochemistry showed that in the immature mouse retina, M6a was strongly expressed in the axons of retinal ganglion cells. After birth, M6a expression was confined to the inner plexiform layer, and finally, to the inner and outer plexiform layers of adult mouse retina. M6a expression was completely paralleled by that of the synaptic marker, synaptophysin. Mouse retinal progenitor cells that overexpressed M6a following retrovirus-mediated gene transfer were subjected to in vitro explant or monolayer cultures. The neurite outgrowth of M6a-overexpressing retinal cells was strikingly enhanced, although M6a did not affect differentiation and proliferation.

**Conclusions:**

These results suggest that M6a plays a role in retinal development by regulating neurites, and it may also function to modulate synaptic activities in the adult retina.

## Introduction

The mature neural retina is organized into a three-layered structure consisting of Müller glia, astrocytes, and six types of neurons. These cells are assumed to differentiate in a precise histogenic order from a single population of multipotent retinal precursors [1]. Various molecules, such as transcription factors and neurotrophic factors, have been reported to play important roles in retinal cell differentiation [2]. However, the intrinsic properties of retinal cells at different developmental stages are still vague. This is in part due to the lack of markers that can identify distinct stages of retinal progenitor cells. In our previous studies, we have tried to identify markers of retinal progenitor cells by employing flow cytometry and cell sorting. Using a panel of antibodies against cell-surface antigens, we screened mouse retinal cells at various developmental stages for reactivity. This technique obtained unique expression patterns for more than 30 antigens in the developing retina. Among them, some CD antigens, such as SSEA-1 (CD15) and c-kit (CD117) were identified as retinal progenitor cell markers in early and late immature stages, respectively [3,4].

Since this approach only identifies known molecules, we used proteomics to examine the comprehensive expression profile of total membrane proteins from embryonic and adult mouse retina. Information about membrane proteins, which are expressed in a specific manner in the developing retina, may not only serve as a tool for purification of retinal subfractions by cell sorting, but may also be useful for analyzing the regulation of retinal development by receptor-signaling and cell surface molecules. To establish such a database, we used shotgun analysis and a nanoflow liquid chromatography-mass spectrometry/ mass spectrometry (LC-MS/MS) system to examine total protein expression in purified membrane fractions [5]. We identified several membrane-associated proteins which are expressed in embryonic retina [5], and among the proteins, we focused on glycoprotein m6a (M6a) in this work.

M6a is a transmembrane protein that belongs to the myelin proteolipid protein (PLP) family. The M6a gene encodes a 278 amino acid protein that contains four putative transmembrane domains with both the N- and C-termini facing the cytosol. PLP constitutes the most abundant protein (approximately 50%) in the central nervous system (CNS) myelin sheath and is involved in signaling through integrins in oligodendrocytes [6]. Although PLP and its splice variant DM20 are glial proteins, M6a is found exclusively in neurons [7]. M6a is present on the postmitotic neurons of the developing neural tube at embryonic day 9 (E9) and is continuously expressed in multiple regions of the CNS in the mouse. Furthermore, the M6a protein is located at the leading edge of the growth cone in cultured cerebellar neurons [8]. Recent studies have suggested the importance of M6a in the process of neural development, such as neurite extension, survival [9], and differentiation [10]. Furthermore, M6a has been found to play an important role in neurite outgrowth and filopodium and spine formation, and may also be involved in synapse formation in cultured hippocampal cells [11]. These findings indicate the possible involvement of M6a in neuronal survival and differentiation. However, the expression pattern and function of M6a in the mouse retina have not been investigated to date.

We identify M6a as a gene that is expressed in the embryonic retina and reveal the expression patterns of M6a in the neural processes, including the nerve fiber layer (NFL), inner plexiform layer (IPL), and outer plexiform layer (OPL), of the immature mouse retina. We also show that the expression of M6a parallels that of synaptophysin. Forced expression of M6a in mouse retinal explant cultures resulted in enhancement of neurite extension, which suggests that M6a plays important roles in the regulation of neurites in the embryonic retina.

## Methods

### Isolation of retina from mice

ICR mice were obtained from Japan SLC, Inc. (Hamamatsu, Japan) and Clea Japan, Inc. (Tokyo, Japan). Mice are housed under 12/12 light/dark cycles in standard shoebox cages with water and food at 23°C. The day that a vaginal plug was observed was considered to be embryonic day 0 (E0), and the day of birth was marked as postnatal day 0 (P0). All animal experiments were approved by the Animal Care Committee of the Institute of Medical Science, University of Tokyo. Mice were euthanized by decapitation or cervical dislocation under anesthesia.

### Plasmid construction and production of retrovirus

The mouse M6a cDNA was cloned by RT–PCR from pooled mouse cDNA from P1 retina. The primers were designed based on the sequences available in the database. A full-length fragment of M6a was cloned into the *Not* I site of the pMXc-IRES-EGFP retrovirus vector (kindly provided by Dr. T. Kitamura, University of Tokyo, Japan), which directs expression of the cloned genes together with enhanced green fluorescent protein (EGFP) from upstream long terminal repeat (LTR) promoter. Production of the retrovirus was performed using the PLAT-E packaging cell line [12] as previously described [13]. Briefly, PLAT-E was transfected with retrovirus vectors containing various genes by using Fugene6 transfection reagent (Roche, Indianapolis, IN) according to the manufacturer’s instructions. Two days after transfection, cell supernatants containing retrovirus were harvested and concentrated by centrifugation in a centrifugal filter device (Millipore, Billerica, MA).

### RT–PCR

Total RNA was purified from mouse retinas by use of Trizol reagent (Gibco BRL, Carlsbad, CA), and cDNA was synthesized with Superscript II (Gibco BRL). The primer sets were tested for several different cycling numbers by using rTaq (Takara, Otsu, Japan), and the semiquantitative cycle number was determined for each primer set. Bands were visualized with ethidium bromide.

### Retinal explant culture, retrovirus infection, and monolayer culture

Retinal explants were prepared as previously described [13]. Briefly, the neural retina was isolated on a Millicell chamber filter and placed with the ganglion cell layer facing upwards. The filters were inserted into six-well plates and cultured in 1 ml of explant culture medium (50% MEM with Hepes, 25% Hank’s solution, 25% heat-inactivated horse serum, 200 mM L-glutamine and 5,75 mg/ml glucose) [13]. Infection of retrovirus was done by exposing the concentrated virus solution to the explant for initial two days, as described previously [14]. Cells were then fixed with 4% paraformaldehyde (PFA) and frozen sectioned. For neurite extension assay, monolayer culture of retina was conducted. Retinal explants were prepared from E17 retinas and infected with retroviruses. After three days in culture, the cells were dissociated by treatment with 0.25% trypsin, and replated on eight-well chamber slides (BD Falcon, Bedford, MA) that were coated with ornithine (Sigma, St. Louis, MO) and fibronrctin (Sigma). The cells were cultured for an additional 11 days in Dulbecco’s modified Eagle’s medium/F-12 medium (Gibco BRL) that was supplemented with 1% fetal bovine serum (JRH Biosciences, Lenexa, KS) and 1% N2 (Gibco). Cells were fixed with 4% PFA and immunostained anti-green fluorescent protein (GFP; Clontech Laboratories, Palo Alto, CA) and anti-glutamine synthetase (GS; Chemicon, Temecula, CA) antibodies. The neurite lengths of GFP-positive and GS-negative cells were examined using Axioplan2 fluorescent microscopy (Carl Zeiss, Oberkochen, Germany). Then, neurite lengths of the cells were measured from randomly taken images using AxioVision 4.6 software (Carl Zeiss, Oberkochen, Germany) and Adobe Photoshop Elements 2 (Adobe Systems, San. Jose, CA). For reaggregation culture, retroviruses were infected into retinal explant cultures prepared from E17.5 retina. After overnight culture, cell were dissociated by treatment with 0.25% trypsin and used as donor cells. They were then mixed with three times number of dissociated retinal cells that had been isolated from the same brood and cultured overnight without virus infection. Then, the aggregates were cultured for eight days, and neurite extension was evaluated after immunostaining with anti-GFP antibody.

### Immunohistochemistry and antibodies

Immunohistochemistry of retinal explants was performed as previously described [4,13]. Briefly, frozen-sections of retinal explant were pre-incubated in a blocking solution containing 2% bovine serum albumin and incubated with the appropriate primary antibody solutions. The primary antibodies and their concentration in reaction solution used were as follows: 1:5,000 dilution anti-GFP polyclonal antibody (Clontech Laboratories), 1:1,000 anti-M6a (clone 321; MBL, Nagoya, Japan), 1:100 anti-Rho4D2 (kindly provided by Dr. R. S. Molday, The University of British Columbia, Vancouver, Canada), 1:1,000 GS (Chemicon), 1:500 anti-Hu C/D (Molecular Probes, Inc., Eugene, OR), 1:100 anti-protein kinase C (PKC; Oncogene Research Product, Boston, MA), and 1:100 anti-Ki67 (BD Biosciences) monoclonal antibodies. The primary antibodies were visualized by using appropriate second antibodies conjugated with 1:1,000 Alexa Fluor 488 or 546. All samples were sealed using VectaShield mounting media (Vector Laboratories, Burlingame, CA) containing DAPI for nuclear staining.

### BrdU labeling and detection

Three days after retrovirus infection, retinal explants were incubated with 5 μM bromodeoxyuridine (BrdU) for 24 h before they were harvested and fixed with 4% PFA. The samples were embedded in optimal cutting temperature (OCT) compound and frozen-sectioned. The sections were treated with 1 U/μl of DNase (Takara) in PBS for 1 h at 37 °C, and the incorporated BrdU was visualized immunohistochemically using an anti-BrdU monoclonal antibody (Roche, Indianapolis, IN) and the appropriate secondary antibodies.

## Results

### M6a is expressed in the neuronal processes of the mouse retina

**Figure 1:**
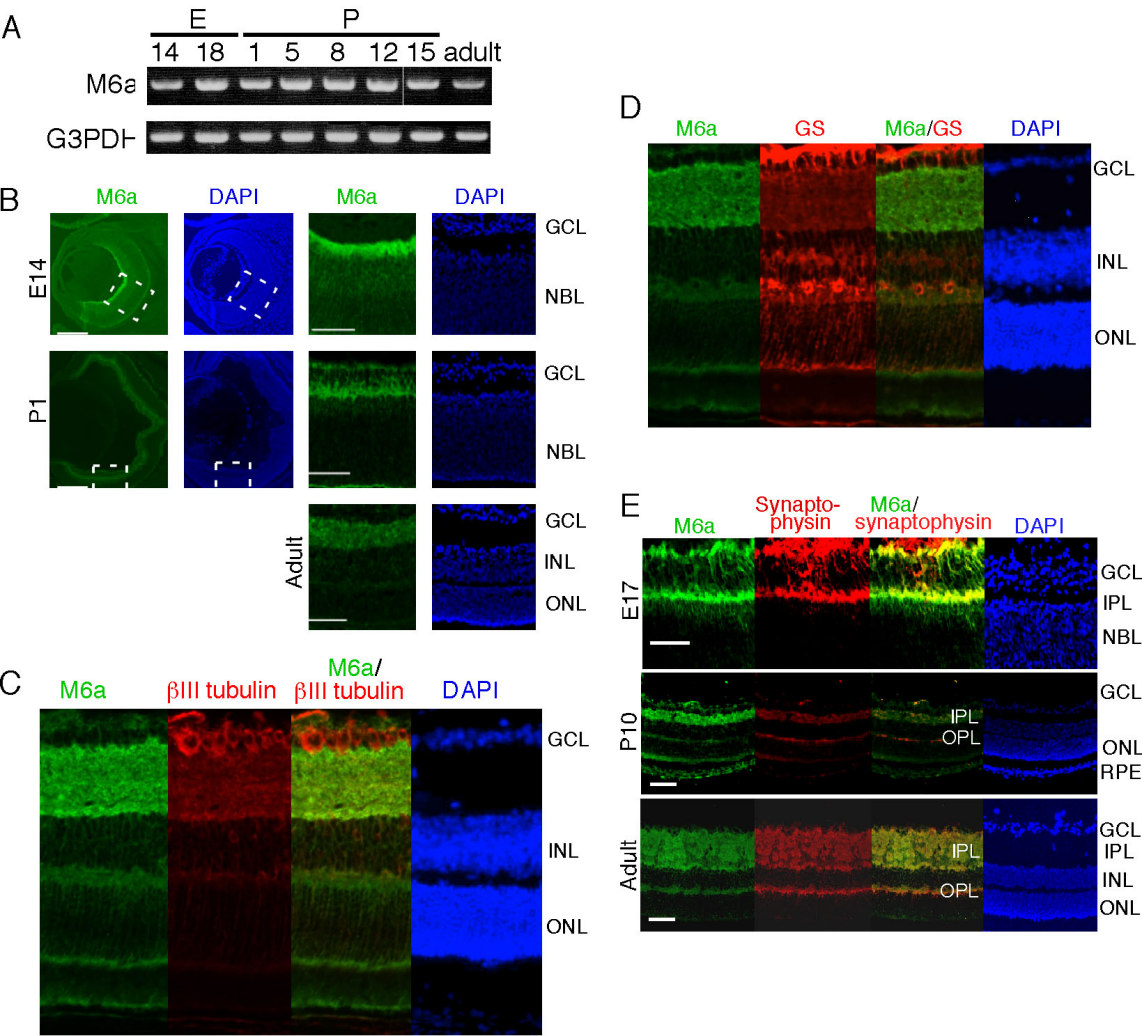
Expression of M6a in mouse retinas from various developmental stages. **A:** Semiquantitative RT–PCR for M6a using total RNA extracted from mouse retinas at various developmental stages. Glyceraldehyde-3-phosphate dehydrogenase (G3PDH) was used as the control. **B-E:** Immunostaining of M6a in frozen sections of mouse retina from various developmental stages. Coimmunostaining was performed with anti-M6a and anti-βIII-tubulin (**C**), anti-glutamine synthetase (**D**), or antisynaptophysin (**E**) antibodies. The right two columns are magnified figure of the square area indicated by broken lines in left two columns **B**. The scale bar represents 100 μm. The following abbreviations are used in this figure: inner plexiform layer (IPL); outer plexiform layer (OPL); ganglion cell layer (GCL); neuroblastic layer (NBL); inner nuclear layer (INL); outer nuclear layer (ONL); retinal pigment epithelium (RPE).

To obtain comprehensive expression profiles of the membrane proteins of embryonic and adult mouse retinas, we analyzed the membrane fractions for total proteins using shotgun analysis on a nanoflow LC-MS/MS system [5]. With this approach, we detected M6a in samples prepared from embryonic retinas, but not in samples from adult retinas [5]. M6a is known to be widely expressed in brain [8], whereas its detailed expression in the neural retinas of mammals has not been reported. We examined the expression of M6a mRNA over time, using semiquantitative RT–PCR (Figure 1A). M6a was expressed in E14 mouse retinas, and expression continued after birth with a slight decrease in the intensities of the bands between P12 and P15. Finally, a weak band was observed in the adult retina samples. We used immunohistochemistry to investigate the spatial and temporal expression patterns of M6a in mouse retina sections from various developmental stages.

In the E14 retina, M6a was mainly and strongly expressed in the NFL, which consists of the axons of ganglion cells, but was not observed in the neuroblastic layer (NBL), which consists of proliferating retinal progenitor cells (Figure 1B). However, in P1 (Figure 1B,C) and P5 (data not shown) retinas, strong expression of M6a was confined to the IPL and also in NFL, which consists of innermost region, but not in ganglion cell layer (GCL). At P10 (data not shown) and in the adult retina, strong expression of M6a was detected in the IPL, and weak signals were observed in NFL, OPL, and inner nuclear layer (INL; Figure 1D).

### M6a protein colocalizes with synaptic markers of postmitotic cells

The βIII tubulin protein is expressed at early stages by differentiated neurons, including ganglion and amacrine cells and by most retinal neurons up to P7 [15]. Double staining of retinal cells with anti-βIII tubulin and anti-M6a antibodies revealed that most cells were double-positive at these stages, which indicates that M6a is expressed on postmitotic mature neurons (Figure 1C). These results are comparable with a previous report that immunolabeling with anti-M6a antibodies was evident throughout the CNS of the embryonic mouse, but was absent from the zones of cell proliferation adjacent to the ventricles [8]. Not only for neurons, but M6a was also weakly expressed in processes of Müller glia cells, which are evident from the co-expression of M6a with Müller glia marker, glutamine synthetase (Figure 1D).

Since previous reports have located M6a immunoreactivity in the synapses of the rat cerebellum and in the axon terminals of the rat cerebellar molecular layer [16], we examined the coexpression of M6a and the presynaptic marker, synaptophysin, by immunostaining with both anti-M6a and antisynaptophysin antibodies (Figure 1E). We found that M6a expression colocalized with that of synaptophysin from the embryonic to adult stages (Figure 1E).

### M6a overexpression does not affect cell fate and subretinal localization of retinal precursors

**Figure 2:**
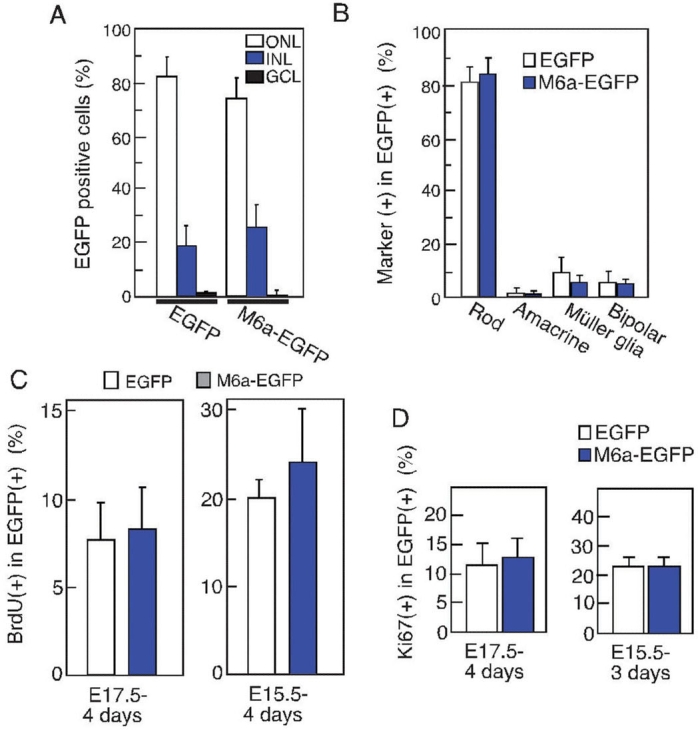
Effects of forced expression of M6a on retinal differentiation and proliferation. **A:** Sublayer distributions of virus-transduced enhanced green fluorescent protein (EGFP)-positive cells in retinal explants. Retinal explants were infected with retorovirus particles that encode M6a and EGFP. After 14 days, the explants were harvested and frozen-sections prepared. Immunostaining was performed using an anti-EGFP antibody. The percentages of cells in each sublayer are shown. More than 200 cells were counted for each sample, and the standard deviation (SD) was calculated from three independent experiments. **B:** Differentiation of virus-infected cells examined by immunostaining to identify subpopulations within the retina. The percentages of marker-positive cells in the EGFP-positive population are shown. Rhodopsin for rod, HuC/HuD for amacrine, glutamine synthetase for Müller glia, and protein kinase C for bipolar were used as markers. More than 100 cells were examined for each sample, and the average value from three independent experiments is shown with the SD. **C**,**D**: Proliferation of M6a-expressing retinal cells was examined by measuring incorporation of bromodeoxyuridine (BrdU; **C**) or expression of the Ki67 antigen (**D**). BrdU was present for the final 24 h of four days of culture of retinal explants, and frozen sections were immunostained using antibodies against BrdU. The same samples were immunostained with the anti-Ki67antibody. The percentage of positive cells with SD are shown. Listed below each panel is the stage when each retinal explant was prepared and its culture period. The following abbreviations are in effect: outer nuclear layer (ONL); inner nuclear layer (INL); ganglion cell layer (GCL).

To examine the biologic significance of M6a for retinal development, we investigated the effects of ectopically expressed M6a in a mouse retinal explant culture prepared from E17, which provides an excellent model to monitor retinal differentiation in vitro [13]. By E17, ganglion cells and a few other cell types have begun to differentiate, and after two weeks in culture, all of the retinal subpopulations have differentiated normally. The mouse retinal explant prepared from E17 was infected with retroviruses that encode either M6a-IRES-EGFP or IRES-EGFP (control) and cultured for two weeks. Since the retrovirus infects only mitotic cells, retinal precursor cells were assumed to be the major targets of gene transfer. We examined the subretinal localization of virus-infected cells. The number of M6a-EGFP-expressing cells was lower in the ONL and slightly higher in the INL than that of control EGFP-expressing retinal cells, but both differences were not statistically significant (Figure 2A). We then examined the differentiation of virus-infected cells by immunostaining frozen sections with antibodies against various marker proteins for retinal cell subpopulations (Figure 2B). The antibodies used were antirhodopsin for rod photoreceptors, anti-HuC/D for retinal ganglion cells, and amacrine cells, anti-PKC for bipolar cells, and anti-GS for Müller glia. The percentage of rhodopsin positive M6a-overexpressing cells was not significantly, but few times higher than that of control cells (Figure 2B). The other retinal cell populations were not affected by M6a overexpression, which suggests that M6a does not play a role in retinal differentiation.

### M6a does not affect retinal precursor cell proliferation

We next examined whether M6a affects the proliferation of retinal cells by measuring BrdU incorporation. Retrovirus-mediated gene transfer into retinal explants prepared from E15.5 or E17.5 was conducted, and proliferating cells were labeled with 5 μM BrdU for the final 24 h of four days of culture. In all cases BrdU incorporation was slightly higher in the M6a-expressing cells than in control cells. Despite this general trend, statistical analysis revealed that the differences were not statistically significant (Figure 2C). Immunostaining the sections with the antiproliferation antigen, Ki67 antibody [17], produced slightly more Ki67-positive cells among the M6a-expressing cells than the control cells. These differences were not considered statistically significant, thus confirming the BrdU results (Figure 2D). Taken together, these results suggested a minor trend of increased proliferation associated with M6a but demonstrated that M6a does not regulate retinal cell proliferation significantly.

To confirm the proliferation results, we performed a clonal assay [4] to test the proliferation capabilities of the virus-transfected M6a cells (data not shown). The colony sizes showed no significant differences between the control and M6a-transfected retinal cells.

### M6a overexpression promotes neurite outgrowth in retina

**Figure 3:**
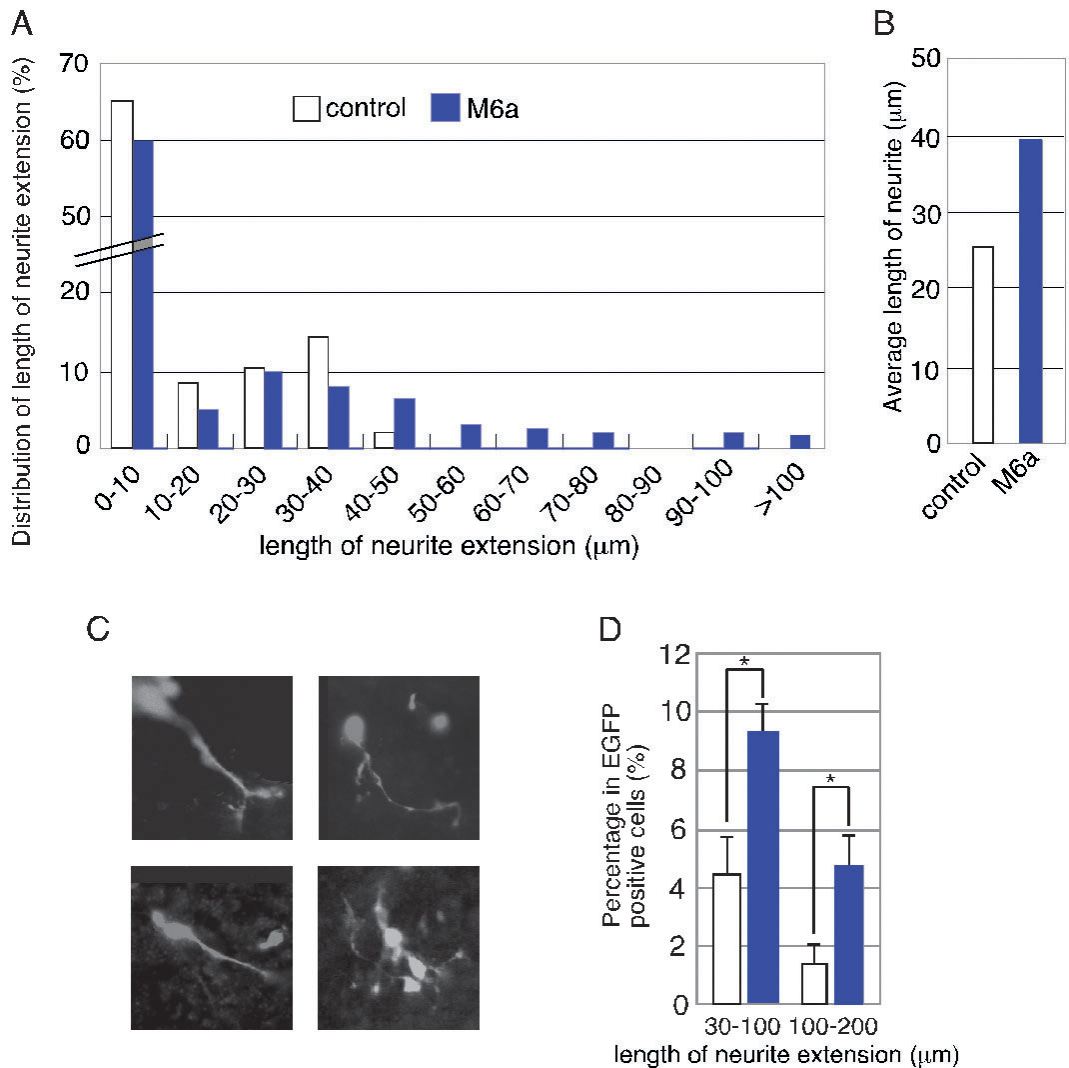
Effects of forced expression of M6a on retinal neurite extension. **A**,**B**: Neurite extension of virus-transduced enhanced green fluorescent protein (EGFP)-positive cells in retinal monolayer cultures. Retinal explants were infected with retorovirus particles that encode M6a and EGFP, and the cells were dissociated and subjected to monolayer culturing. After 11 days of culture, the cultures were harvested and stained with anti-GFP antibody. Neurite length was examined by measuring the EGFP-positive neurites on photographs taken under a fluorescence microscope. The length distribution percentages for the neurites are shown in **A**. The average lengths of neurites longer than 10 μm are shown in **B**. More than 100 cells were counted for each sample, and essentially the same results were obtained in three independent experiments. **C**,**D:** Neurite extension of virus-transduced EGFP-positive cells in retinal reaggregation cultures. Morphology of the M6a-EGFP virus-transfected retinal cells in reaggregation culture (**C**). The percentage of cells with neurite extensions of 30-100 μm or 100-200 μm in the M6a and control EGFP expressed retinal cells (**D**). Asterisk is p<0.05 by the Student's *t*-test.

Since M6a has been implicated in neurite extension in brain, we investigated whether M6a was also involved in neurite extension in the retina, using monolayer cultures of retinas infected with retroviruses that encode control EGFP or M6a-IRES-EGFP. Neurite extension in monolayer cultures was examined by measuring the length of neurites from photographs taken under a fluorescence microscope. To distinguish neural cells from glial cells, we stained the retinal cells with antibody against GS, which is a glial cell marker, and evaluated the neurite lengths of the GS-negative and EGFP-positive cells. In both the control and M6a-expressing samples, approximately 60% of the cells extended neurites by 0–10 μm of neurite. However, when we compared the cell population distribution categorized by neurite length, we discovered that M6a-expressing cells extended longer neurites than did control virus-infected cells (Figure 3A). The average neurite lengths were greater than 10 μm for the M6a-overexpressing cells (39.2 μm) and control cells (25.5 μm; Figure 3B). We next confirmed these results with a different method of culture. We prepared reaggregation cultures by mixing dissociated virus-infected retinal explants prepared from E17.5 and dissociated retinal cells isolated from the same brood. Eight days later, percentage of M6a-overexpressing retinal cells bearing neurite over 30 μm length was about twice-times higher than that of control EGFP expressing cells (Figure 3C,D). This indicates that M6a plays a role in promoting the neurite extension during retinal development.

## Discussion

### M6a protein is targeted to the neuron processes of the murine retina

In the present study, strong expression of M6a was detected in the ganglion cell axons and processes of cells located in the INL of the immature murine retina. A previous report on in situ hybridization of M6a in the *Xenopus* eye demonstrated expression of M6a mRNA in the INL and GCL, and weak expression in the ONL [18], which suggests that the expression of M6a in the retina is conserved in vertebrates.

In mouse hippocampal tissues, M6a mRNA has been found to be expressed in granule cells of the dentate gyrus and pyramidal neurons in CA1 and CA3; immunoreactivity for M6a was concentrated in the regions of relatively dense synaptic contact [11]. Similar results have been obtained for the rat [16]. This implies that the translated M6a protein is targeted to processes distal to the somata. The expression of M6a paralleled that of the presynaptic protein, synaptophysin, in the mouse retina, suggesting that the subcellular distribution of M6a is the same in the brain and retina. It has been established that synapse formation by dissociated neurons in culture strongly correlates with focal accumulations of structures that can be labeled with antibodies against synaptic vesicle proteins, such as synaptophysin [19]. Therefore, our results indicate a possible role for M6a in the formation of synapses in neural retinas.

### M6a does not affect retinal progenitor cell differentiation and proliferation

We found that cell fate and subretinal localization of retinal progenitor cells were not affected by M6a overexpression. To date, there has been no report on the promotion of proliferation by M6a. On the other hand, it has been reported that the addition of anti-M6a antibody decreases the survival of dissociated neurons in culture and inhibits the extension of neurites in cultured cerebellar explants [9]. M6a belongs to the proteolipid protein (PLP)/DM20 family of myelin proteins. PLPs play a pivotal role in early oligodendrocyte differentiation and survival [20]. PLP members have been observed to form a complex with integrins and may participate in integrin receptor signaling in oligodendrocytes [21]. Given the high degree of homology between M6a and PLP, it is possible that this transmembrane protein is also related to integrin signaling. Our preliminary results from examining integrin family proteins in the developing retina reveal the expression of integrin αv in the embryonic mouse retina (unpublished results) which suggests that M6a plays role in retinal development through interaction with integrins to mediate signals for retinal development.

### M6a promotes neurite outgrowth in the retina

In the present study, we observed promotion of retinal neurite extension by M6a. This protein has been reported to enhance neurite extension in rat pheochromocytoma PC12 cells and to induce an increase in the intracellular Ca^2+^ concentration of PC12 cells [10]. The anti-M6 antibody efficiently interfered with Ca^2+^ influx, which suggests that M6a acts as a Ca^2+^ channel blocker in PC12 cells. As a second messenger, Ca^2+^ has been shown to participate in neurite extension, filopodium and spine activity, and neuronal differentiation [22]. M6a possesses two PKC phosphorylation sites. Treatment of PC12 cells with a PKC inhibitor eliminates the ability of M6a to promote nerve growth factor-primed neurite extension [10]. Thus the putative phosphorylation sites in the cytosolic domains may facilitate M6a regulation and may be relevant for intracellular signaling. Therefore, the promotion of neurite extension by M6a in retinal cells seen in the present study is possibly associated with Ca^2+^ influx, and phosphorylation of PKC may remove this function. Future studies will focus on this hypothesis.
